# Incidence and Risk Factors for Delirium among Mechanically Ventilated Patients in an African Intensive Care Setting: An Observational Multicenter Study

**DOI:** 10.1155/2015/491780

**Published:** 2015-04-05

**Authors:** Arthur Kwizera, Jane Nakibuuka, Lameck Ssemogerere, Charles Sendikadiwa, Daniel Obua, Samuel Kizito, Janat Tumukunde, Agnes Wabule, Noeline Nakasujja

**Affiliations:** ^1^Department of Anaesthesia, Makerere University College of Health Sciences, Mulago National Referral Hospital, P.O. Box 7051, Kampala, Uganda; ^2^Department of Internal Medicine, Mulago Hospital Complex, Mulago Hill, P.O. Box 7051, Kampala, Uganda; ^3^Department of Clinical Epidemiology and Biostatistics, Makerere University College of Health Sciences, Mulago National Referral Hospital, P.O. Box 7051, Kampala, Uganda; ^4^Department of Psychiatry, Makerere University College of Health Sciences, Mulago National Referral Hospital, P.O. Box 7051, Kampala, Uganda

## Abstract

*Aim*. Delirium is common among mechanically ventilated patients in the intensive care unit (ICU). There are little data regarding delirium among mechanically ventilated patients in Africa. We sought to determine the burden of delirium and associated factors in Uganda. *Methods*. We conducted a multicenter prospective study among mechanically ventilated patients in Uganda. Eligible patients were screened daily for delirium using the confusional assessment method (CAM-ICU). Comparisons were made using *t*-test, chi-squares, and Fisher's exact test. Predictors were assessed using logistic regression. The level of statistical significance was set at *P* < 0.05. *Results*. Of 160 patients, 81 (51%) had delirium. Median time to onset of delirium was 3.7 days. At bivariate analysis, history of mental illness, sedation, multiorgan dysfunction, neurosurgery, tachypnea, low mean arterial pressure, oliguria, fevers, metabolic acidosis, respiratory acidosis, anaemia, physical restraints, marital status, and endotracheal tube use were significant predictors. At multivariable analysis, having a history of mental illness, sedation, respiratory acidosis, higher PEEP, endotracheal tubes, and anaemia predicted delirium. *Conclusion*. The prevalence of delirium in a young African population is lower than expected considering the high mortality. A history of mental illness, anaemia, sedation, endotracheal tube use, and respiratory acidosis were factors associated with delirium.

## 1. Introduction

Delirium is especially common among mechanically ventilated patients and is associated with worse outcomes such as increased length of stay, higher costs of care, prolonged mechanical ventilation, and a higher mortality [1–3]. Numerous studies have incidences varying from 11 to 80% [3–5] depending on study population differences, methodology, and nature of study. ICU delirium as a concept is new in the African setting. With regard to Uganda, few ICU attending physicians are aware of it and as such its assessment is not routinely performed in this setting. In spite of the risks and outcomes, the number of mechanically ventilated patients is increasing due to increasing access to critical care even in low-income settings, placing more patients at risk for the aforementioned adverse outcomes and delirium [1, 6].

To the best of our knowledge, there is no data regarding delirium among mechanically ventilated patients in African intensive care units. We sought to determine the incidence, outcomes of delirium, and associated factors among patients admitted in Ugandan ICUs.

## 2. Materials and Methods

We conducted a prospective multicenter observational study in 4 intensive care units in Kampala, Uganda, for 18 months from 2013 to mid-2014. Mulago Hospital is a 1500-bed facility with a twelve-bed ICU previously described in the literature [7]. International Hospital Kampala, Case Hospital, and Kadic Hospital are private hospitals in the capital of Kampala with combined 20 ICU beds. All ICUs use the closed model, and are staffed by anaesthesiologists and internists. They nurse to patient ratio on average is 1 : 2. They handle a case mix of patients from all over the country since they comprise 90% of the country's ICU capacity. Services offered include mechanical ventilation and intermittent haemodialysis. Eligible participants were expected (according to the admitting intensivist) to require mechanical ventilation for at least 48 hours. We excluded patients who were younger than 18 years old, patients who were brain dead, patients whose next of kin refused to consent, those paralyzed at time of assessment, and those extubated before day of initial assessment. Patients who were sedated were monitored against the Richmond Agitation-Sedation Scale (RASS) of −3 to 0 to maintain arousable state. The clinical teams decided on sedation regimen and routes of administration and whether administration was by bolus or infusion. All other managements were at the discretion of the ICU staff.

### 2.1. Delirium Screening

Various study tools are available for assessing delirium [8]. We chose the confusional assessment method (CAM-ICU) tool for our study due to its proven use and translatability across all ICU patients [9–11]. Briefly, CAM evaluates the four key delirium features: (1) acute onset and fluctuating course, (2) inattention, (3) disorganized thinking, and (4) altered level of consciousness. Delirium is considered present if features (1) and (2) were present in addition to either feature (3) or (4). Before the start of the study, 2 study assistants (ICU nurses) received training to explain the aim of the study and use of the study tools for delirium detection. The study assistants gave oral and written information about the study and delirium in general to attendants of patients admitted to the ICU. Upon obtaining informed consent, all patients were screened daily for delirium at the bedside using the CAM-ICU. To conduct the inattention (feature 2) part of the CAM-ICU in patients who had difficulty with the alphabet, numbers were used (ten random numbers where the number 3 was repeated four times). In assessing for inattention, the patients were asked to squeeze the assessor's hand once they heard the number 3, in any language. This was practiced once with the patient. The assessor then mentioned a string of 10 numbers with 4 of them being “threes” and 6 of them being other single digit numbers. We deemed the patient to be inattentive if he/she got less than 8 correct squeezes or nonsqueezes.

The tool was administered daily during the study period (day or night). CAM-ICU was conducted if patients were in a RASS range of −2 to +1 (lightly sedated). We classified the patient as delirium positive or not. Patients were followed from ICU admission until death or discharge. Patients were considered coma and delirium free if they had a RASS above −3 with a negative CAM-ICU score and were exited from the study. Administration details and dosage of all sedative, analgesic, and adjunct medications (intravenous infusion or bolus) were collected daily.

### 2.2. Data Collection and Outcome Measures

We collected patient demographics at enrollment. Medicosociohistory included smoking history, psychiatric history, alcohol intake, education level, modified early warning score (MEWS), and acute physiological and chronic health evaluation (APACHE). Clinical characteristics included organ dysfunction, surgical status, comorbidities, and biochemical and haematological values. Treatment characteristics included sedation drugs, use of restraints, endotracheal intubation, mode of ventilation, chest X-ray findings, and route of administration of feeds. Primary outcome was presence of delirium. Secondary outcomes were death and length of ICU stay.

### 2.3. Ethical Considerations

The study was approved by the Research and Ethics Committee of the School of Medicine, College of Health Sciences, Makerere University, as well as the Uganda National Council of Science and Technology according to the ethical standards laid down in the 1964 Declaration of Helsinki and its later amendments. All study participants or their next of kin gave informed consent to participate in the study.

### 2.4. Statistical Analysis

To be able to detect with 95% power a 10% difference in incidence in our hospitals from the previously reported incidence in hospitalized patients who required mechanical ventilation [1], a sample size of 160 was required. All data were analyzed using STATA (StataCorp. 2011.* Stata Statistical Software: Release 12*. College Station, TX: StataCorp LP).

Categorical data has been presented as percentages and continuous data as means with standard deviations. Comparisons were made between participants who developed delirium and those who did not. For the categorical variables, Pearson *χ* ^2^ tests or Fisher's exact test was used. For the continuous data, Student's *t*-test or Wilcoxon rank sum test was used for the normally and skewed data, respectively. Unadjusted logistic models were used to examine the effect of various factors independently on delirium presence (age, gender, admission diagnosis, history of mental illness, history of alcohol intake, length of ICU stay, use of restraints, type of airway, sedation schedule, drugs, and metabolic abnormalities). Variables with *P* < 0.20 were included in a multivariable logistic regression model. The level of statistical significance was set at *P* < 0.05.

## 3. Results

There were 160 eligible participants assessed for delirium. The mean age was 36.6 years; the majority was male and had some level of education (Table 1). The commonest admission diagnosis (50%) was traumatic brain injury (TBI) followed by ARDS (acute respiratory distress syndrome) 16.8% (Table 2). Patients were mostly on pressure-controlled ventilation (PCV) and had tracheostomies (Table 3). The main mode of feeding was enteral. The mean APACHE score was 27 (Table 4). Overall ICU/hospital mortality was 69% (Table 3). Delirium was in 81/160 (50.94%) and resolved in 32% of sufferers among survivors (Table 3). Predictors of delirium at bivariate analysis (Table 5) were a history of mental illness (0.043), having multiorgan dysfunction (*P* = 0.04), neurosurgery (*P* = 0.01), a high respiratory rate (*P* = 0.01), a higher average PEEP (0.001), a higher mean arterial pressure (MAP) (*P* = 0.03), oliguria (*P* = 0.01), fevers (*P* = 0.01), acidosis (*P* = 0.03), hypercapnia (*P* = 0.01), anaemia (*P* = 0.01), sedation (*P* = 0.01), and endotracheal tube use (*P* = 0.009) (Table 3). Use of tracheostomy, not having had surgery, and having no sedation seemed to be protective. At multivariable logistic regression, patients who had a history of mental illness, endotracheal tubes, anaemia, respiratory acidosis, a higher average PEEP, and sedation were likely to have delirium (Table 5).

There was no association between delirium and mortality.

## 4. Discussion

This multicenter prospective cohort study was conducted to determine the incidence of delirium among mechanically ventilated patients in an African setting. Our study is the first to explore delirium among critically ill patients in an African population.

We determined that delirium was present in 51% of mechanically ventilated patients, in agreement with multiple studies conducted in other settings [1, 2, 4, 12]. This incidence maybe low taking into account the high APACHE score reported in our study. This is due to several reasons. Firstly, a single daily assessment was performed; this meant that delirium by its nature, a fluctuating condition, was missed on some occasion. Secondly, a delirium expert or psychiatrist did not always monitor our study assistants to ensure strict assessment of RASS/delirium. Lastly, we were unable to either stratify for the depth/severity of delirium nor measure the duration of delirium in those that recovered, so we may have missed “hypoactive delirium.”

The patient demographics in our study are similar to what has been previously reported for this setting [7]. The high APACHE scores in our study possibly arose from the demand for the few ICU beds in our setting and therefore relatively higher mortality than that reported in the literature. A significant majority of our study population were young single males, who sustained traumatic brain injuries (TBI) following motorcycle road traffic accidents. The commonest reason for intensive care admissions as had been found in other low-income country ICUs is traumatic brain injuries sustained during road traffic accidents, a common feature in this setting [7].

In this study, delirium screening was performed using the CAM-ICU because it has been previously validated in mechanically ventilated patients and has good predictive value [1, 13]. The CAM-ICU has been found to have good interrater reliability and is easily translatable as reported from numerous studies [8, 9, 11, 14]. With regard to the use of numerals for analphabetic patients, we left this to the discretion of the study nurses who found it much easier to perform than the standard tool. In addition, we did not plan to look at differences with literate patients and therefore did not document this. However, a vernacular based study is commencing and we plan to validate this with a hypothesis that the use of numbers maybe more sensitive compared to the use of words in the general population.

At bivariate analysis, notable predictors of delirium were a positive history of mental illness, having multiorgan dysfunction, neurosurgery for traumatic brain injuries, fevers, acidosis, hypercapnia, anemia, sedation, and endotracheal tube use. Many of these have been documented as predictors of delirium in other studies [2, 4, 5, 7, 15–17].

At multivariate analysis, however, a history of mental illness, endotracheal tubes use, anaemia, respiratory acidosis, having a higher PEEP, and having had sedation were associated with delirium in agreement with other studies [4, 5, 18, 19].

The use of endotracheal tubes is an independent predictor of delirium and maybe associated with higher sedation requirements [20] and as earlier observed use of sedatives was associated with delirium.

Sedation is given to ensure comfort and to minimize distress but is linked to delirium and immobility [21]. Early deep sedation has been associated with adverse outcomes [22]. Furthermore, a strategy of no sedation in critically ill patients undergoing mechanical ventilation has been reported to result in fewer mechanical ventilation days and shorter ICU/hospital length of stay compared to a standard sedation strategy [6, 23].

We were unable to document sedation dosage among patients with endotracheal tubes though benzodiazepines were the only intermittent sedatives given in this setting.

Benzodiazepines have high affinity for gamma-aminobutyric acid (GABA_A_) receptors, whose activation can alter levels of numerous neurotransmitters believed to be deliriogenic [21]. Additionally, they impair the quality of sleep via slow-wave sleep suppression, thus possibly contributing to delirium [24, 25]. Opioids common in our setting such as morphine, tramadol, remifentanil, and fentanyl, alternatives to benzodiazepines, have offered better outcomes [26–28].

Patients with anemia (Hb less than 10 g/dL) were likely to suffer delirium in our study. Low hemoglobin levels have been reported along with low oxygen saturation and low haematocrit as being part of the oxidative stressors linked to delirium [18].

Our study shows that higher PEEPs were associated with development of delirium. At multivariate analysis, we noted that ARDS has a weak association with the development of delirium. A possible association between mechanical ventilation and delirium has been attributed to aberrant vagal sensory induced apoptosis in that brain area within 90 min of commencing MV without causing hypoxia, oxidative stress, or inflammatory responses [2].

The association between the development of delirium and respiratory acidosis in this study is similar to what has been reported in physiologic and psychiatric studies in both healthy subjects and critically ill patients [29, 30].

In this study, delirium resolved in 32% of sufferers. This was probably due to the use of haloperidol or modification of risk factors by the attending physicians. Haloperidol is one of antipsychotics used to treat delirium [24] but studies have not supported its use as prophylaxis [31].

There was no relationship between delirium and mortality in our study but we believe the study was not prospectively powered to determine a definitive relationship between delirium and mortality. Moreover, this finding is similar to what was reported in a recent meta-analysis looking at randomized interventions to treat delirium [32].

Our study had several limitations in addition to its observational nature. Due to the method of record keeping, we were unable to accurately document sedation dosages.

We did not study interrater reliability of the CAM-ICU. However, during the study preparatory phase adequate training and practice on the instrument were achieved. Our study was not designed to prove a cause-and-effect relationship between delirium and clinical outcomes and since we only followed patients until death or discharge, our mortality analysis was not as comprehensive as previous studies that followed patients for longer periods.

## 5. Conclusion

Our study is the first to report delirium among African ICU patients. The study was conducted in 4 busy tertiary medical centers, which included a broad range of patients with both medical and surgical conditions. We believe our admission diagnoses represent demographics of most busy ICUs in Africa; however, this may not apply to higher income settings. Additionally the associated factors found in this study have also been previously reported in the literature. While the incidence may have been deceptively low due to limitations in study protocol, it does not mask the fact that there is a need to implement staff education and patient focused strategies as well as prevention of delirium especially in a resource limited setting.

## Figures and Tables

**Table 1:** Baseline demographic characteristics.

Variable	Categories	No delirium	Delirium	*P* value
		*N* (percentage)	*N* (percentage)	
Sex	Male	50 (47.62)	55 (52.38)	0.239
	Female	20 (37.04)	34 (62.96)	

Marital status	**Single**	**23 (33.33)**	**46 (66.67)**	**0.009**
	**Married**	**47 (54.65)**	**39 (45.35)**	

Education level	None	7 (30.43)	16 (69.57)	0.336
	Some	62 (53.13)	75 (46.87)	

Mental illness history	**Yes**	**11 (68.75)**	**5 (31.25)**	**0.036**
	**No**	**59 (41.26)**	**84 (58.74)**	

Alcohol intake history	Yes	13 (38.24)	21 (61.76)	0.614
	No	47 (43.12)	62 (56.88)	

Smoking history	Yes	19 (54.29)	16 (45.71)	0.166
	No	51 (41.13)	73 (58.87)	

**Table 2:** Baseline admission characteristics.

Variable	Categories	No delirium	Delirium	*P* value
		*N* (percentage)	*N* (percentage)	
Admission diagnosis	ARDS	12 (44.44)	15 (55.56)	0.056
	Cardiac	12 (70.59)	5 (29.41)	
	TBI	32 (39.02)	50 (60.98)	
	Sepsis	4 (25.00)	12 (75.00)	
	Polytrauma	9 (56.25)	7 (43.75)	

Multiorgan dysfunction	**TBI**	**6 (50.00)**	**6 (50.00)**	**0.000**
	**HIV**	**24 (70.59)**	**10 (29.41)**	
	**Diabetes**	**4 (40.00)**	**6 (60.00)**	
	**AKI**	**6 (100.00)**	**0 (00.00)**	
	**ARDS**	**6 (21.43)**	**22 (78.57)**	
	**Sepsis**	**6 (18.18)**	**27 (81.82)**	

Urine output	**Normal**	**50 (53.46)**	**43 (46.24)**	**0.003**
	**Oliguria**	**20 (30.30)**	**46 (69.70)**	

Temperature	**Normal**	**44 (55.00)**	**36 (45.00)**	**0.005**
	**Fevers**	**26 (32.91)**	**89 (55.97)**	

PH	Normal	10 (27.78)	26 (72.22)	0.273
	Acidosis	37 (40.66)	54 (59.34)	

Hemoglobin	**Normal**	**28 (70.00)**	**12 (30.00)**	**0.000**
	**Anemia**	**42 (35.29)**	**77 (64.71)**	

Creatinine	Normal	53 (44.54)	66 (55.46)	0.822
	High	17 (42.50)	23 (47.50)	

Urea	Normal	44 (49.44)	45 (50.56)	0.392
	High	21 (38.18)	34 (61.82)	

**Table 3:** ICU treatment characteristics.

Variable	Categories	No delirium	Delirium	*P* value
		*N* (percentage)	*N* (percentage)	
Sedation	**Sedation **	**23 (28.40)**	**58 (71.60)**	**0.000**
	**None **	**47 (60.26)**	**31 (39.74)**	

Sedative given	Benzodiazepine	34 (37.36)	57 (62.64)	0.183
	Propofol	11 (55.00)	9 (45.00)	
	Opioid	19 (51.35)	18 (48.65)	

Endotracheal tube	Yes	54 (48.65)	57 (51.35)	0.112
	No	16 (34.78)	30 (65.22)	

Tracheostomy tube	Yes	15 (33.33)	30 (66.67)	0.072
	No	55 (49.11)	57 (50.89)	

Restraints	**Yes**	**10 (20.41)**	**39 (79.59)**	**0.000**
	**No**	**60 (55.05)**	**49 (44.95)**	

Mode	PVC	26 (44.07)	33 (55.93)	0.584
	VCV	21 (42.00)	29 (58.00)	
	SIMV	20 (52.63)	18 (47.37)	

Enteral feeding	Good	48 (41.03)	69 (58.97)	0.203
	Not good	22 (52.38)	20 (47.62)	

Outcome	Discharge	21 (42.86)	28 (57.14)	0.843
	Death	49 (44.55)	61 (55.45)	

**Table 4:** Clinical characteristics of patients with and without delirium by univariate analysis.

Variable	Delirium (*n* = 89)		No delirium (*n* = 70)		*P* value
	Mean	SD	Mean	SD	
Age	35.202	14.170	38.429	16.215	0.183
APACHE II	27.360	6.231	25.686	6.437	0.506
Heart rate	104.191	20.764	99.000	20.253	0.116
Respiratory rate	**23.247**	**7.146**	**20.143**	**6.212**	**0.003**
Mean arterial pressure	**79.011**	**25.886**	**70.914**	**18.667**	**0.029**
Blood sugar	8.439	2.196	8.349	1.867	0.786
PO_2_	69.697	27.179	62.329	36.142	0.144
PCO_2_	**48.258**	**15.611**	**40.200**	**19.781**	**0.005**
SPO_2_	90.854	9.794	87.429	20.077	0.160
HCO_3_^−^	**18.546**	**4.995**	**16.701**	**5.450**	**0.028**
White blood cell	14.666	7.071	16.086	10.032	0.297
GCS	7.888	2.846	7.686	3.142	0.672
FiO_2_	55.539	27.269	53.214	25.876	0.586
Length of stay	9.878	8.907	8.843	6.792	0.464

**Table 5:** Significant predictors for delirium among ICU patients on mechanical ventilation at bivariate and multivariate analysis.

Variable	Bivariate analysis			Multivariate analysis		
	OR ratio	95% CI	*P* value	OR ratio	95% CI	*P* value
Mental illness history	3.132	1.034–9.488	0.043	0.18	0.05–0.59	**0.005**

Multiorgan dysfunction						
TBI	1	—		1	—	
HIV	0.42	0.11–1.61	0.204	0.52	0.08–3.38	0.493
DM	1.50	0.27–8.19	0.640	2.65	0.18–38.82	0.477
HTN	3.00	0.42–21.30	0.272	4.16	0.31–55.60	0.281
ARDS	3.67	0.86–15.59	0.079	6.44	0.86–48.30	0.070
Sepsis	4.50	1.07–18.92	0.040	3.64	0.51–25.76	0.196
Had neurosurgery	0.419	0.217–0.810	0.010			

Higher respiratory rate	1.076	1.024–1.130	0.004			
Higher MAP	1.016	1.001–1.030	0.031	1.01	0.98–1.04	0.574
Oliguria	2.674	1.376–5.199	0.004	1.94	0.54–6.89	0.307

Fevers	2.491	1.309–4.742	0.005			

Respiratory acidosis	1.027	1.008–1.046	0.006	1.04	1.01–1.08	**0.018**
Metabolic acidosis	1.071	1.007–1.139	0.030	1.07	0.94–1.21	0.312
Anaemia	4.278	1.973–9.274	0.000	5.65	1.57–20.36	**0.008**
Sedation	0.262	0.135–0.507	0.000	0.18	0.05–0.59	**0.005**
PEEP	1.345	1.120–1.616	0.002	1.38	1.04–1.83	**0.024**
Endotracheal intubation	1.78	0.87–3.62	0.114	6.89	1.65–28.71	**0.008**

Surgery	0.42	0.22–0.81	0.010	0.29	0.07–1.22	0.092

## Key Messages

This paper demonstrates the presence of delirium among African ICU patients. Factors associated with delirium in this setting are similar to those in higher income countries. Awareness regarding delirium must be initiated among staff in African ICUs.
